# Toward Responsible and Privacy-Preserving Generation of Inpatient Clinical Discharge Notes Using Large Language Models

**DOI:** 10.34133/csbj.0151

**Published:** 2026-07-09

**Authors:** Felice Burn, Chantal Zwick, Joseph Weibel, Cyril Fischer, Rick Veer, Lennart Essmann, Maurin Lampart, Michael Chamo, Carola George, Sandra Gafner, Simone Wildisen, Martin Vanecko, Kimberly Ordoñez, Monica Alexandra Vieira, Christian Eisenring, Beat Mueller, Daniel Olivier Perruchoud, Tristan Struja

**Affiliations:** ^1^ Center of Excellence for AI and Data Science, Cantonal Hospital Aarau, Aarau, Switzerland.; ^2^ Institute for Data Science, FHNW Brugg-Windisch, Windisch, Switzerland.; ^3^ University Department, Division of General Internal and Emergency Medicine, Cantonal Hospital Aarau, Aarau, Switzerland.; ^4^ Notfall Zurich 3, NZ3, Zurich, Switzerland.; ^5^ Laboratory for Computational Physiology, Institute for Medical Engineering and Science, Massachusetts Institute of Technology, Cambridge MA, USA.; ^6^ Hospital Muri,Aargau,Switzerland.

## Abstract

Writing discharge notes (DNs) is a time-intensive task in inpatient care that contributes substantially to physician workload and burnout. Large language models (LLMs) offer potential opportunities to automate clinical documentation. However, concerns regarding accuracy, reliability, data privacy protection laws and data governance, and compliance with healthcare privacy remain. This study evaluated the performance of locally deployed, domain-adapted LLMs for generating German-language DN in internal medicine. In this retrospective study, electronic health record data from a Swiss tertiary care hospital were used. Clinical information from 3 inpatient scenarios served as input for the 3 open source LLMs (Mixtral 8×7B, Mixtral 8×22B, and LLaMA 3.1 70B). Zero-shot prompting, 4-shot in-context learning, and supervised fine-tuning using QLoRA were compared. Generated DNs were evaluated using automated metrics, blinded physician assessment with direct comparison of all relevant clinical information using the modified Physician Documentation Quality Instrument, and additionally an LLM-as-a-judge framework and a Turing Test. Across all evaluation methods, the fine-tuned Mixtral 8×7B model achieved the best overall performance compared with prompting-based approaches and larger nonspecialized models. Simpler cases consistently received higher-quality ratings. Physician inter-rater agreement was low, highlighting the challenges of objective quality assessment for clinical documentation. In a Turing Test, physicians were unable to reliably distinguish artificial intelligence-generated from clinician-authored DNs, performing at chance level, demonstrating that high-quality DN generation is feasible using locally deployed LLMs within secure hospital infrastructures. Domain-specific fine-tuning substantially improves performance and may enable practical deployment. Future work should address further hallucination mitigation, robustness across medical specialties, and real-world integration.

## Introduction

Administrative tasks such as writing discharge notes (DNs) represent a substantial burden for clinicians. They spend up to half of their workday interacting with electronic health records (EHRs) [1]. Deterrence from bedside tasks has been closely linked to rising rates of burnout and reduced job satisfaction [1]. The time-consuming task of writing DN is essential for ensuring safe transitions of care integrating complex and multidisciplinary diagnostic and therapeutic decisions and communicating clear follow-up plans for providers.

Despite their critical role in care continuity, DNs are mostly written by junior staff and vary in quality. Poorly written DNs have been associated with adverse outcomes, including medication errors, hospital readmissions, and missed outpatient appointments [2–4]. Automating the generation of DN could substantially improve documentation efficiency while supporting safer patient handovers and potentially increase the time spent at bedside.

Although natural language processing (NLP) with large language models (LLMs) offers great possibilities for automation, many LLMs are not specifically and extensively trained on medical texts [5]. English-speaking teams demonstrated that LLMs can create DNs that are comparable to clinician-authored DNs [6], or even outperform junior clinicians in content provision and clarity [7].

However, several challenges remain before LLMs can be responsibly deployed in clinical routine [8]. These include factual inaccuracies (so-called hallucinations), failure to capture key diagnostic nuances, lack of generalizability across clinical domains, and important concerns regarding data privacy and data governance, especially in the demanding regulatory context of the European Union [9]. Previous studies found that hallucinations were present in 40% of ChatGPT-generated psychiatric DNs, some of which were clinically relevant [10] or even harmful [6,9]. While LLMs have been evaluated on English medical texts, their application to non-English languages remains limited, with particularly few studies addressing German medical data. Evaluating models that are deployable within local IT environments is also essential to ensure compliance with health data protection regulations [11].

These challenges motivate the investigation of locally deployable LLMs. This study focuses specifically on technical and qualitative evaluation in a controlled setting of locally hosted LLMs tasked with generating German DNs based on documented clinical inputs from EHRs.

## Methods

We performed a retrospective cohort study at the Cantonal Hospital Aarau, a tertiary, 500-bed hospital in Switzerland, with approximately 6,000 annual medical admissions. All admissions to the Medical University Clinic between October 2015 and June 2024 were eligible as long as patients did not actively reject the general research consent.

The evaluation focused on 3 common inpatient scenarios that differ in clinical complexity and documentation style: planned chemotherapy (PC), acute coronary syndrome (ACS), and acute lower back pain (ALBP) (Fig. 1). Board-certified physicians assessed the quality of the generated DN compared with all necessary clinical information of the clinical information system needed to write a human-written DN. We defined a complex case as having 300 words or more, or more than 2 diagnoses, or more than 1 intervention during the hospitalization. We further examined whether the generated texts could be reliably distinguished from manually authored DNs by a Turing Test.

**Fig. 1.**
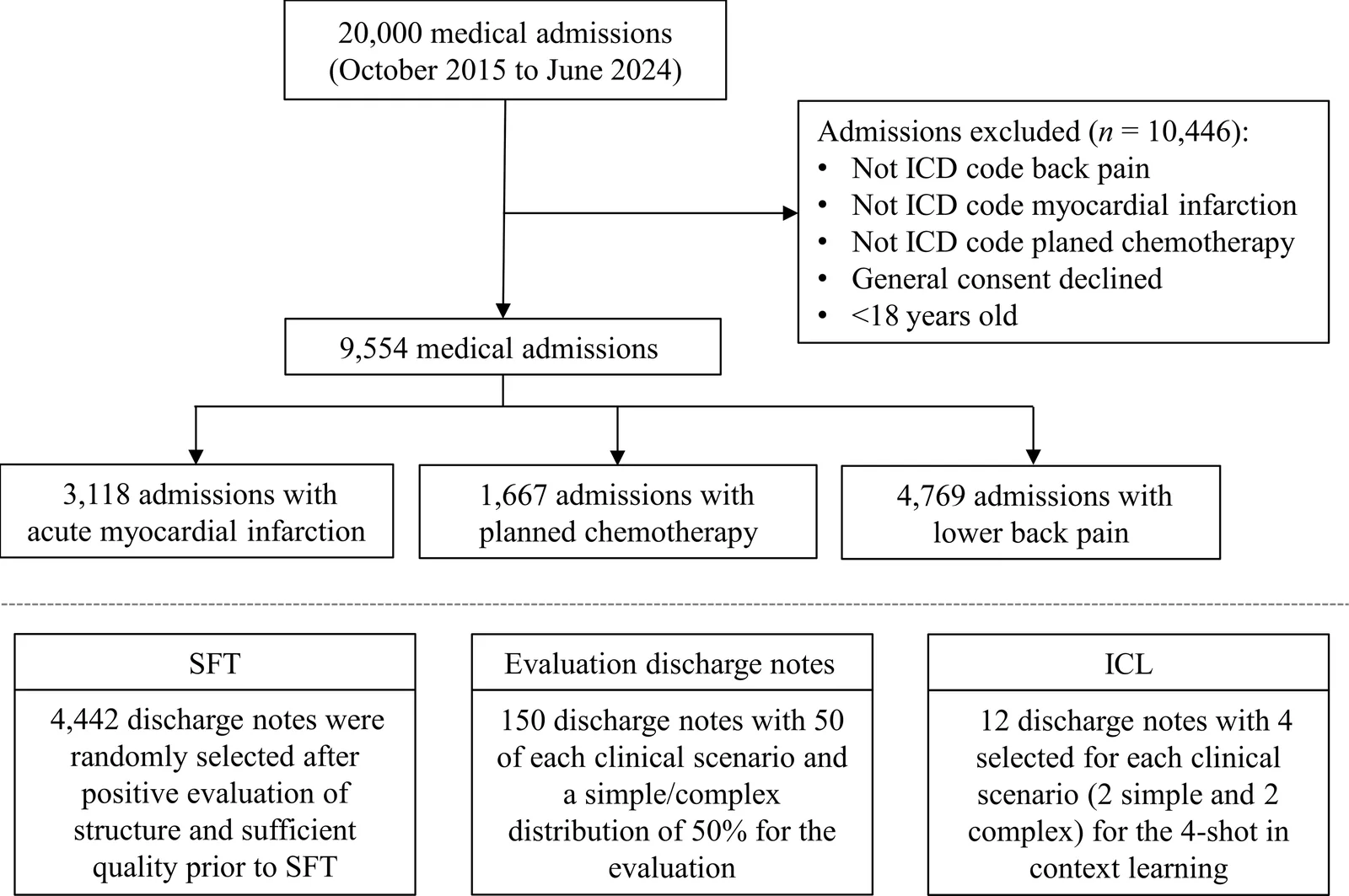
Flowchart: selected patients, exclusions, and further distribution of the cohort.

The Ethics Review Board of Northwestern Switzerland approved the study including the waiver for individual informed consent (EKNZ #2022-00977). We only extracted information from the hospital’s EHR of the clinic information system (KISIM Cistec) for 3 clinical scenarios: (a) PC, (b) ACS, and (c) ALBP. Cases were identified by ICD-10-GM codes.

All text was pseudonymized using a internally validated algorithm detecting and replacing patient identifiable information (PII) [12]. Then, admission and discharge dates (ADD), history of present illness (HPI), diagnoses and procedures (DXL), and the corresponding human-authored DNs were retained as the gold standard validation data. The pipeline received the input data to the LLM as JavaScript Object Notation (JSON) format. All data extraction, processing, and modeling were carried out in an on-premises, secure hospital infrastructure to comply with data privacy regulations.

### Model selection and configurations

We evaluated 3 decoder LLMs of varying parameter sizes and architectures: Mixtral 8×7B, Mixtral 8×22B [13], and LLaMA 3.1 70B [14]. The LLMs were selected based on German language support and state-of-the-art performance at that time.

All models were initiated with the same prompt, enforcing the desired format and clinical tone of the DN (Supplementary Materials; for the English translation, see Fig. S1, and for the original German version, see Fig. S2). The prompt was iteratively refined through expert feedback during the study setup.

Three prompting configurations were tested:

• Zero-shot prompting: Mixtral 8×7B received only ADD, HPI, and DXL inputs, along with instructions.
• 4-Shot prompting: Each of the 3 models were prompted with corresponding, high-quality DN examples for PC, ACS, and ALBP. These examples served as reference outputs of DN to guide a model’s in-context learning (ICL). Four examples were hand-picked by a senior physician for each of the 3 clinical scenarios, 2 simple and 2 complex cases each.
• Supervised fine-tuning (SFT): Only Mixtral 8×7B was fine-tuned separately for each clinical scenario using the QLoRA method [15]. Training dataset sizes were PC (*n* = 1,028), ACS (*n* = 1,920), and ALBP (*n* = 1,494). An internal validation set of 10 samples per use case was used for early stopping of training. Detailed hyperparameters and code are provided in the accompanying GitLab repository.

All prompting configurations used temperature value [16] set to 0 decoding next tokens by highest probability (Fig. 2).

**Fig. 2.**
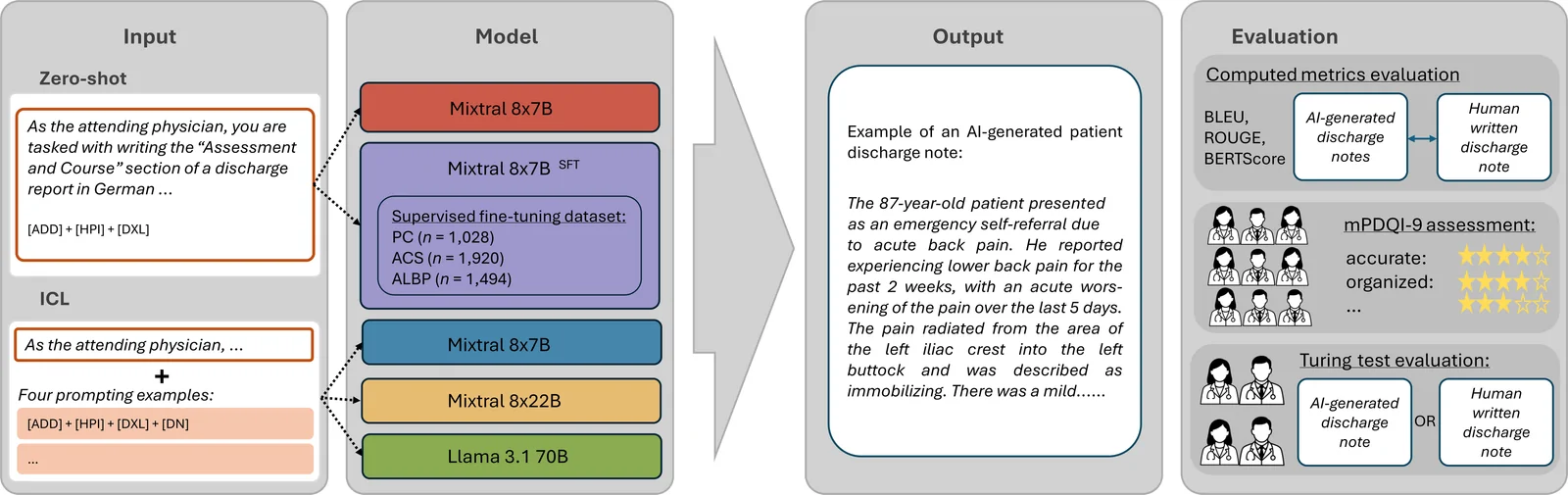
Schematic study design. Input prompts in English (original German prompts with corresponding translation in Fig. S1). ADD, admission and discharge dates; HPI, history of present illness; DXL, diagnoses and procedures.

### Data privacy compliance

Models were 4-bit quantified for inferencing locally on a secure hospital infrastructure with 2 Nvidia RTX 6000 ADA GPUs (48 GB each) respecting compliance within institutional data protection standards and national standards due to data privacy legislation and compliance with the national human research act (HRA). This design mitigated the risk of any potential exposure of sensitive health data to external providers and aligns with responsible artificial intelligence (AI) deployment in healthcare settings based on actual regional legislation rules of national data protection laws, the Human Research Act, and the Declaration of Helsinki.

### Test dataset

For rater evaluation, we randomly selected DNs as follows. For each of the 3 clinical scenarios, 5 simple and 5 complex samples were randomly selected, resulting in 30 unique records. These were used to generate DNs with 5 different combinations of LLM models and prompting configurations resulting in 150 variations of generated DNs according to Fig. 1. Because of the resource-intensive nature of multiexpert clinical evaluation, a subset of 30 cases was selected, enabling detailed assessment across multiple model configurations and evaluation frameworks while maintaining feasibility.

### Evaluation

The generated DNs were quantitatively evaluated using metrics widely used in previous evaluations of medical summarization tasks.

• BLEU (Bilingual Evaluation Understudy) [17] and ROUGE (Recall-Oriented Understudy for Gisting Evaluation) [18], to assess n-gram overlap (Python package evaluate);
• BERTScore [19], which uses contextual embeddings to measure semantic similarity between generated and ground truth (human-authored) texts (Python package bert-score).

The generated DNs were additionally evaluated by 9 physicians based on a modified version of the Physician Documentation Quality Instrument (mPDQI-9) [20], a validated tool for the assessment of the quality of clinical documentation. Our adapted MPDQI-9 instrument comprises 9 important text quality domains: internal consistency, syntheticism, organization, succinctness, thoroughness, usefulness, accuracy, comprehensiveness, and free of hallucinations, each rated on a 5-point Likert scale (1 = poor, 5 = excellent). Scores are summed across domains, resulting in a total score ranging from 9 to 45. Raters were also asked additionally to give an overall expression ranging from 0 to 10. For both assessments, higher points indicated better performance. Raters were blinded to model identity and prompting configurations. Written instructions were provided for calibration purposes of the raters. Ratings were summed per rater for each summary, yielding a total quality score ranging from a minimum of 9 to a maximum of 45 points. Inter-rater reliability was assessed using Krippendorff’s alpha and Kendall’s *W*. We used the implementation validated in Ref. [21] available at https://www.k-alpha.org and Kendall’s *W*, which measure the concordance in overall consistency across the 9 raters. For both measures, values close to zero indicate no agreement, whereas values close to one indicate strong agreement. In addition, pairwise binary agreement was computed to capture how often 2 raters agreed in preferring the output of one model over another. For each rater pair, this metric reflects the proportion of cases in which both raters deemed the same model as superior.

As an additional evaluation method, we employed an additional LLM to rate the DN using the same mPDQI-9 score as the human experts (LLM-as-a-Judge). To avoid self-enhancement bias, we selected Qwen 2.5 72B [22], which was not used in generating any of the summaries. The model received blinded input pairs and returned structured ratings for each of the 9 mPDQI-9 indicators, enabling direct comparison with human-generated scores.

To assess the distinguishability of human-written and AI-generated DNs, a Turing Test was set up with the best performing model (SFT) results in accordance with Turing’s original formulation. The test assesses whether a human evaluator can reliably distinguish between human and machine responses [23].

The independent external raters were grouped into 2 senior and 2 junior general practitioners to investigate the consistency between the evaluators participating. To each rater, 60 DNs were presented, containing 30 written by humans and 30 that were AI-generated. For each DN, the rater had to decide if it was an AI-written discharge summary or not. We computed inter-rater reliability using Krippendorff’s alpha and performed a 2-sided binomial test to observe classification accuracy against chance-level performance.

## Results

### Quantitative evaluation

Performance across BLEU, ROUGE-L, and BERTScore (F1) metrics varied notably depending on the model, prompting configuration, and case complexity. Among all configurations, the SFT of Mixtral 8×7B consistently achieved the highest scores across all 3 metrics. Detailed results are provided in Table 1.

**Table 1.** Computed evaluation metrics (ranging from 0 to 1) for LLM-generated DN. Columns “Simple” and “Complex” distinguish between the 2 complexity levels of the cases. Boldface indicates the best-performing model and values.

Model	Variant	BERTScore (F1)		BLEU		ROUGE-L	
		Simple	Complex	Simple	Complex	Simple	Complex
Mixtral 8×7B	Zero shot	0.686	0.704	0.017	0.026	0.128	0.146
Mixtral 8×7B	4-shot	0.720	0.707	0.068	0.048	0.209	0.176
Mixtral 8×7B	SFT	**0.754**	**0.744**	**0.183**	**0.129**	**0.346**	**0.269**
Mixtral 8×22B	4-shot	0.718	0.719	0.078	0.083	0.214	0.197
LLaMA 3.1 70B	4-shot	0.735	0.720	0.109	0.084	0.277	0.206

The Mixtral 8×7B SFT model significantly outperformed all other models, including second best option LLaMA 3.1 70B with ICL across BERTScore (*P* = 0.01, Wilcoxon signed-rank test), BLEU (*P* < 0.001), and ROUGE-L (*P* < 0.001) independent of the complexity of the patient case and input information.

### Human evaluation

In addition to automated evaluation, all 150 discharge summaries were assessed by 9 physicians (5 fellows and 4 senior physicians) using the modified mPDQI-9 scale. Figure 3 presents the distribution of the summation of the rater scores across model and prompting configurations.

**Fig. 3.**
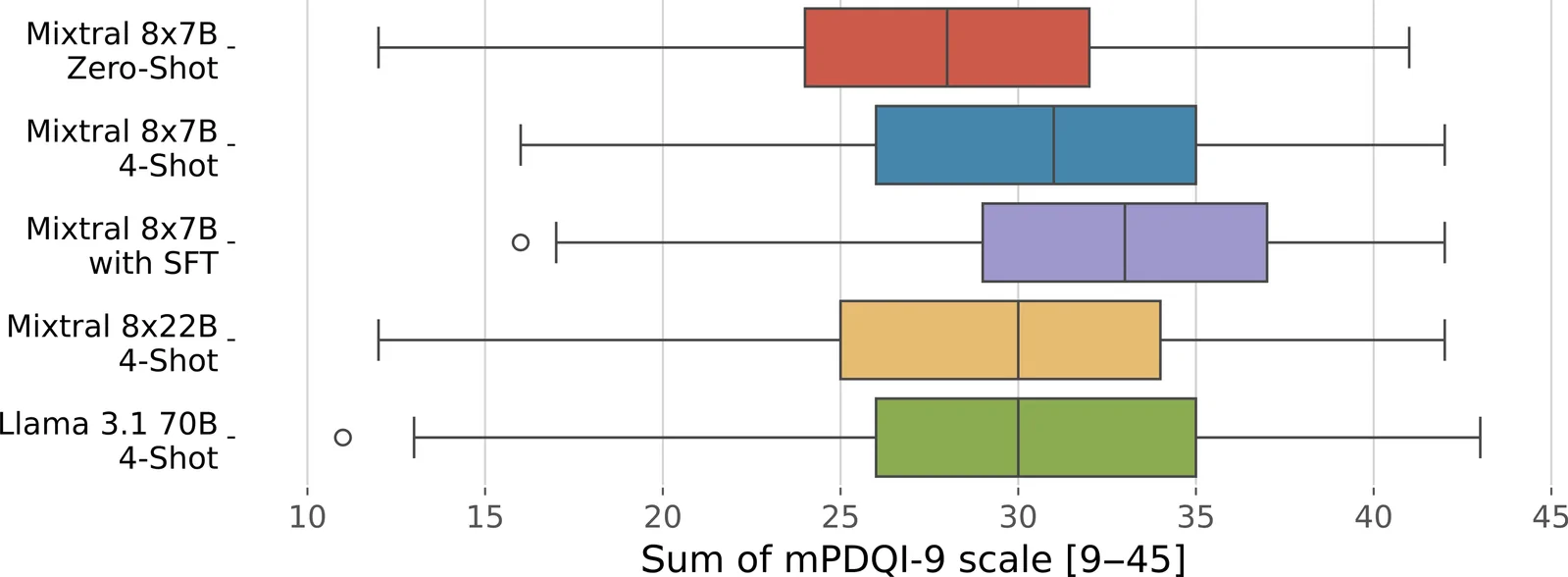
Distribution of mPDQI-9 scores (ranging from 9 to 45) and median values across human raters for all model and prompting configurations, *n* = 270 per model and prompting configuration.

Similar to the calculated metrics, raters gave the highest mPDQI-9 median score of 33 to the Mixtral 8×7B with SFT. Mixtral 8×7B with 4-shot refining yielded a modest improvement with a median of 32 over zero-shot prompting of Mixtral 8×7B with a median of 28. The larger model Mixtral 8×22B performed with a median of 30 similarly to its smaller counterpart Mixtral 8×7B while LLaMA 3.1 70B had a similar median score but exhibited the widest range of scores by the 9 raters.

The mPDQI-9 rating evaluation according to the 3 clinical scenarios revealed that the PC scenario benefited the most from SFT (Fig. 4) compared to the other 2 scenarios.

**Fig. 4.**
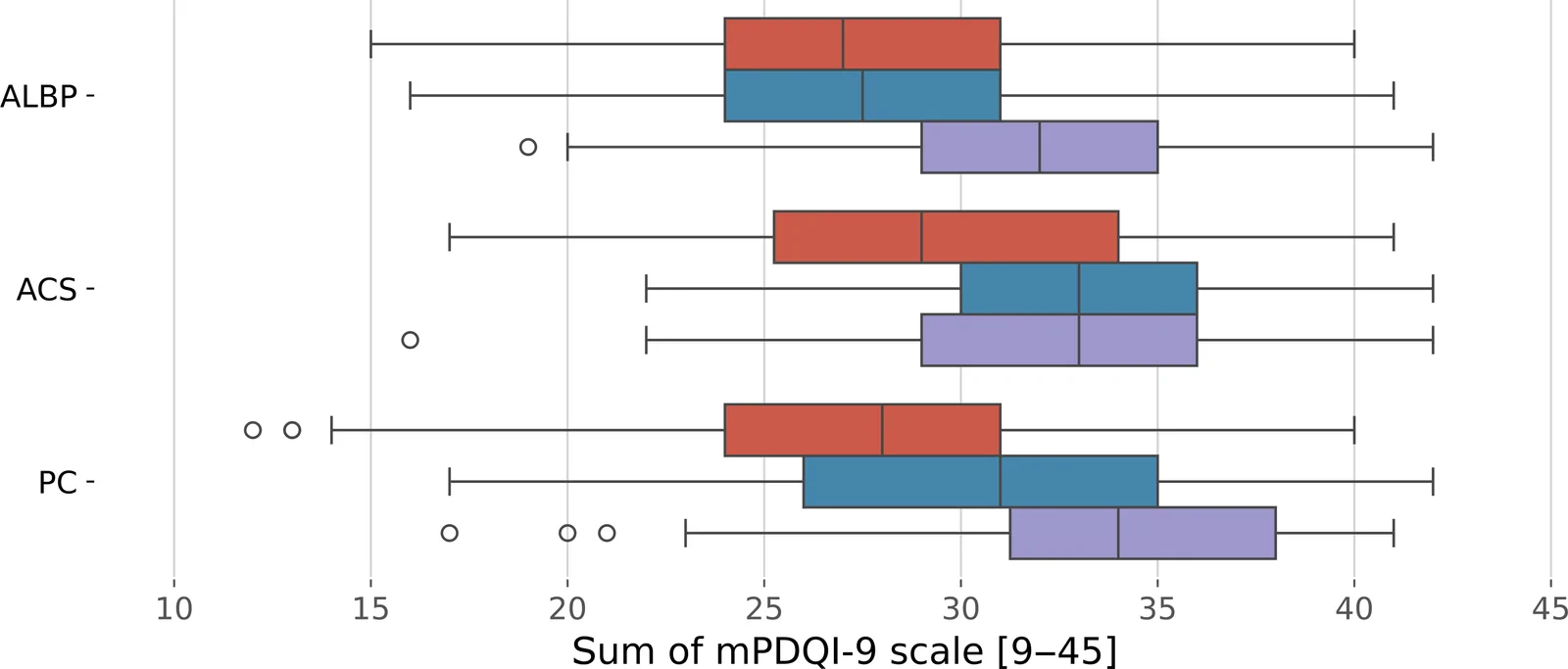
Distribution of mPDQI-9 scores (ranging from 9 to 45) across model configurations for different clinical scenarios of planned chemotherapy (PC), acute coronary syndrome (ACS), and acute lower back pain (ALBP) with the Mixtral model series (red: Mixtral 8×7B with zero-shot, blue: Mixtral 8×7B with ICL, and purple: Mixtral 8×7B with SFT), *n* = 90 ratings per model and scenario.

The rater scoring between case complexity across all models and configurations is seen in Fig. 5. DNs generated for simple cases received a median rating that was 3 points higher than those for complex cases. Figure 6 presents the mean response quality scores for each model across the 9 indicators of the mPDQI-9 instrument (the Supplementary Materials provide additionally mean response quality scores grouped into fellows [Fig. S3] and seniors [Fig. S4]). Notably, the supervised fine-tuned model (Mixtral 8×7B with SFT) outperformed other configurations across nearly all dimensions. It achieved the highest ratings in key areas such as comprehensible, organized structure, less synthetic tone, and internal consistency in the generated DN, while internal consistency was relatively similar across all models.

**Fig. 5.**
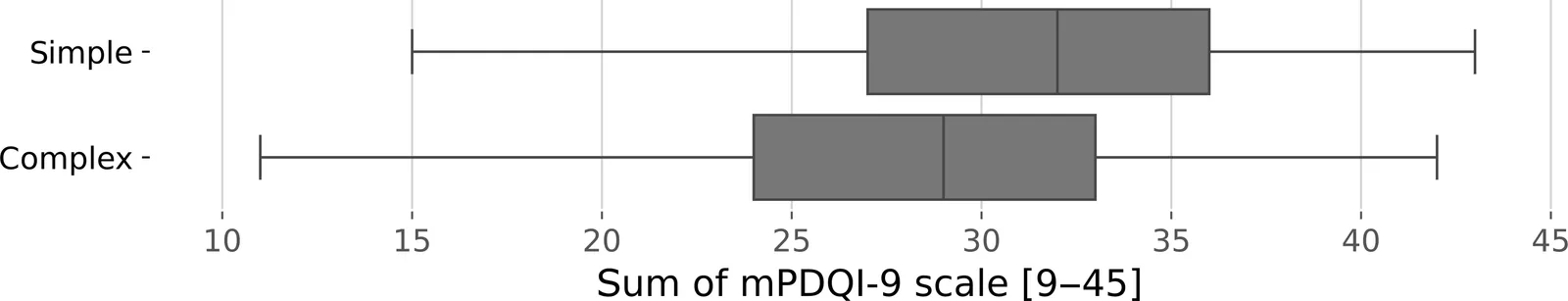
Distribution of mPDQI-9 scores stratified by case complexity, *n* = 675 per group.

**Fig. 6.**
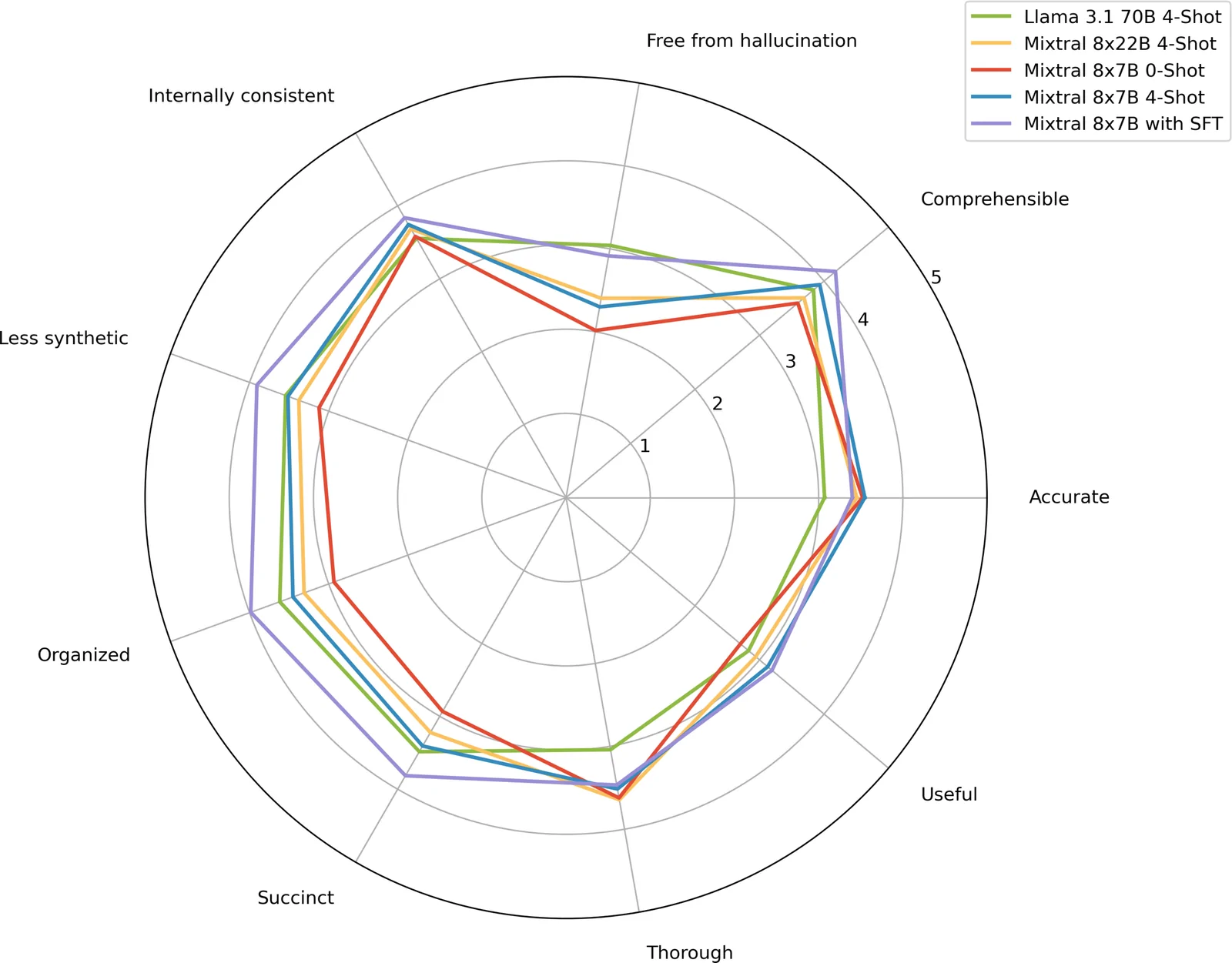
Mean scores of mPDQI-9 scale by question type and for each model, *n* = 270 per group.

In contrast, other models particularly those with zero- or few-shot prompting demonstrated more variability and underperformed in stylistic criteria. For example, Mixtral 8×7B zero-shot lagged behind on metrics related to organization and less synthetic responses.

### Inter-rater agreement

Despite the use of a standardized assessment tool, inter-rater agreement among the 9 physicians was low. Krippendorff’s alpha indicated poor agreement (alpha = 0.326; 95% CI: [0.247, 0.392]), and Kendall’s *W* suggested low concordance in ranking model outputs (*W* = 0.009; 95% CI: [0.007, 0.011]).

To further explore rating consistency, a pairwise binary agreement was computed (see Fig. 7). These numbers represent the proportion of cases in which 2 raters independently selected the same model as superior in a direct comparison, and an agreement level of 50% indicates random preference. Agreement rates varied substantially between rater pairs ranging from 62% to 74%.

**Fig. 7.**
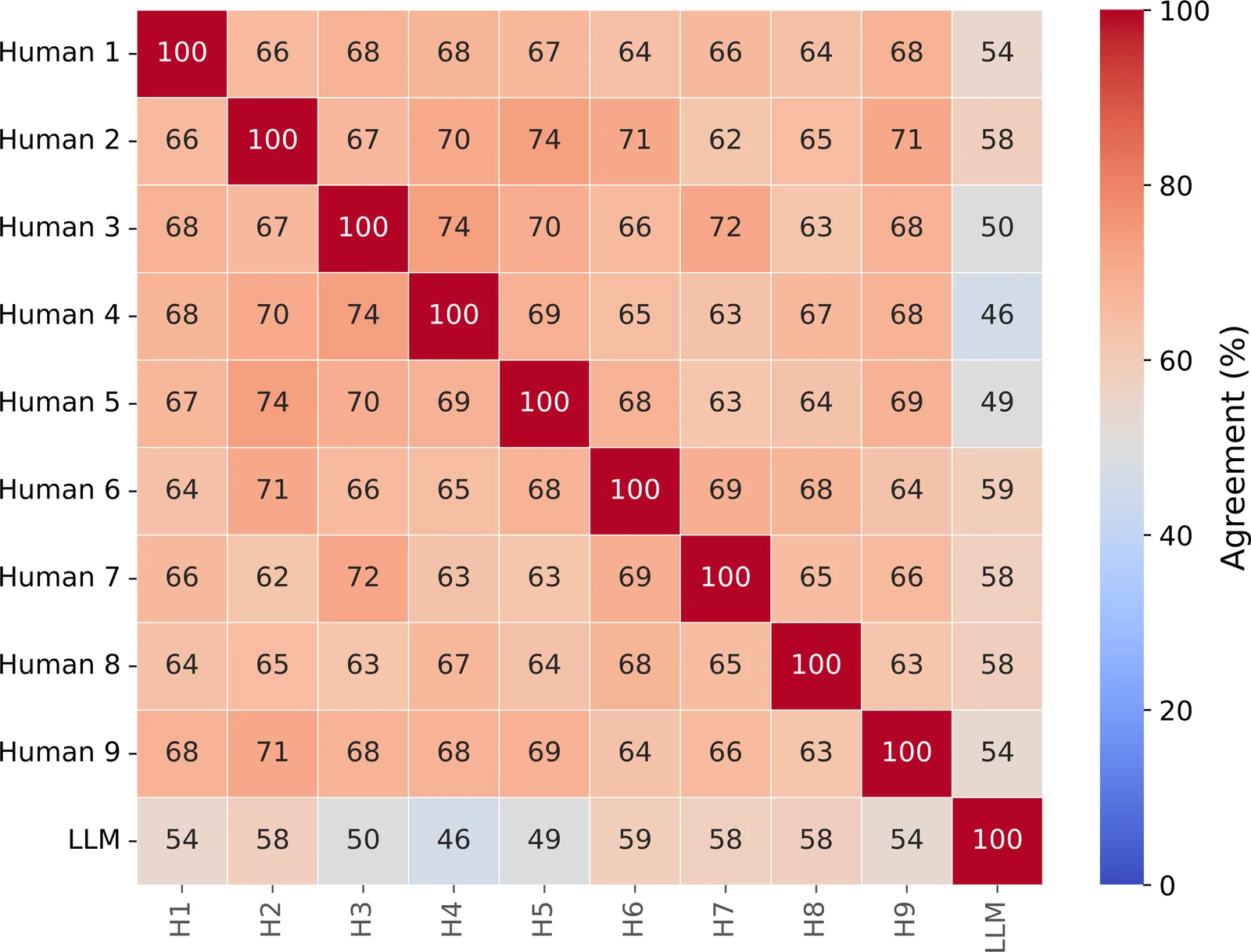
Pairwise binary agreement level (0 to 100) between 9 human raters (H1 to H9) and LLM-as-a-judge (LLM Qwen 2.5 72B).

Furthermore, agreement scores involving LLM-based judge were consistently lower than those by the raters, ranging from 46% to 58%.

### Turing Test with referral physicians

To assess the distinguishability of the generated DNs from the human-written DN by referring physicians, a Turing Test was established.

Figure 8 displays the resulting combined confusion matrix evaluation for all raters. The confusion matrix over all raters shows an accuracy of 51.7% (95% CI: [45.4%, 57.9%]), a precision of 52.5% (95% CI: [41.7%, 63.1%]), a sensitivity of 35.0% (95% CI: [27.1%, 43.9%]), and a specificity of 68.3% (95% CI: [59.6%, 76.0%]). The inter-rater agreement was poor (Krippendorff’s alpha = 0.0913), and the 2-sided binominal test for chance-level performance after Bonferroni correction is *P* = 0.62. The confusion matrix of each involved rater is available in Figs. S5 to S8.

**Fig. 8.**
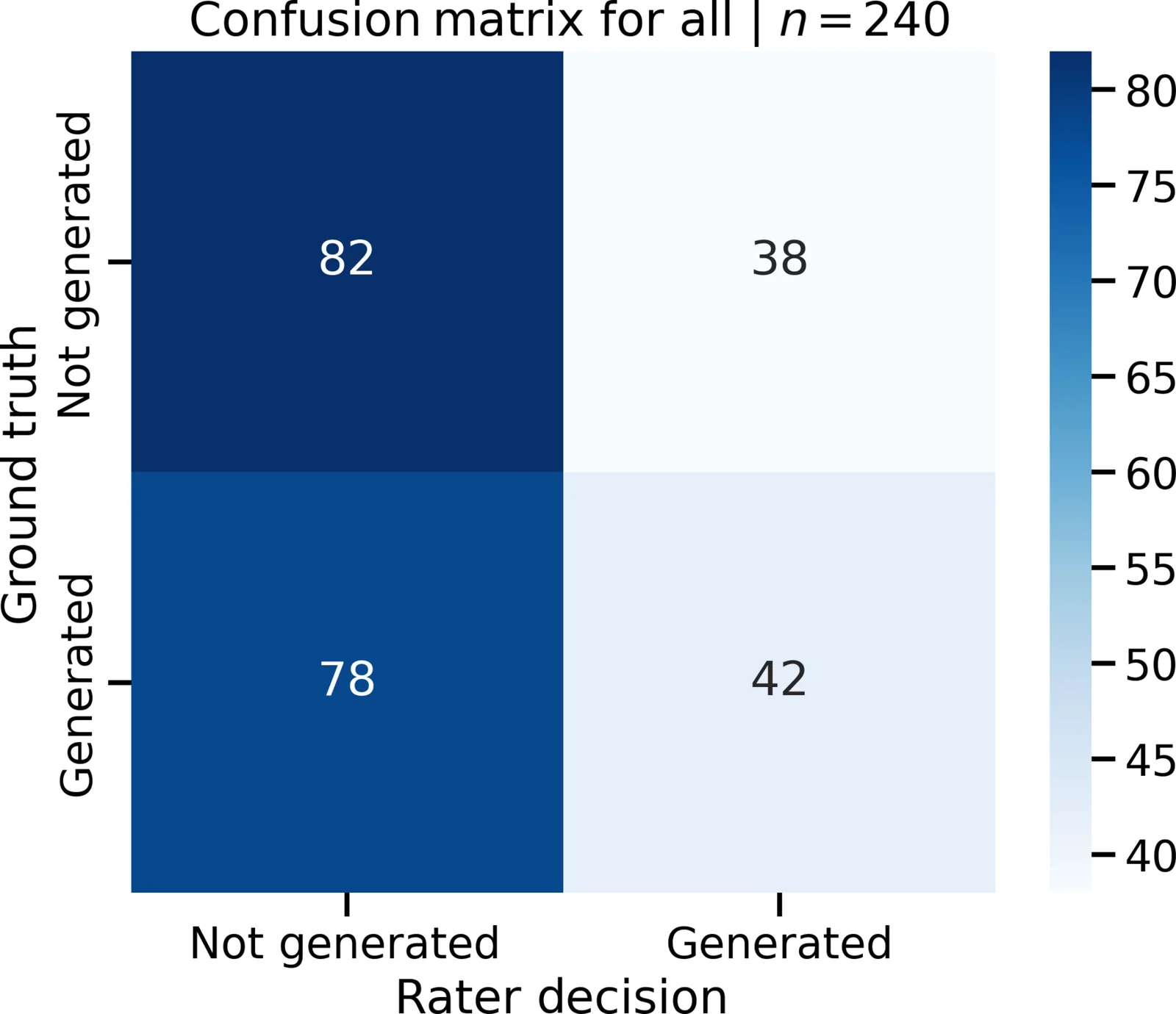
Turing Test: 2 fellow and 2 senior physicians distinguishing AI-generated from human-authored DN.

## Discussion

This study evaluated the capability of open-source LLMs to generate DN based on structured clinical inputs in a controlled, retrospective setting and on a local deployed infrastructure. Among the models and configurations tested, the Mixtral 8×7B with SFT consistently outperformed all other approaches across both automatic evaluation metrics (BERTScore, BLEU, and ROUGE-L) and human ratings, underscoring the importance of domain-specific adaptation and structured prompting for improving model performance in clinical documentation tasks. Even though lexical overlap metrics such as BLEU and ROUGE have limited ability to reflect clinically meaningful differences between model outputs, our consistent findings across such metrics and expert-based evaluations (mPDQI-9) strengthen the validity of the multidimensional evaluation framework.

The 9-physician panel (including 4 seniors and 5 fellows) evaluated each of the 150 model-generated notes using the mPDQI-9 instrument, focusing on aspects such as clinical accuracy, clarity, structure, hallucinations, and completeness. These assessments revealed that the SFT model not only achieved the highest overall quality scores, but also consistently outperformed across most individual mPDQI-9 dimensions, as illustrated in the spider diagram in Fig. 6. Notably, its superiority was most pronounced in stylistic and structural categories such as comprehensibility, structural organization, and synthetic tone reduction—a finding that suggests that fine-tuning allows models to better replicate the professional style and internal logic of clinical narratives. Compared to the other Mixtral models with or without ICL, the fine-tuned Mixtral 8×7B showed less rater-identified inconsistencies in the hallucinations assessment similar to Raunak *et al.* [24]. The fine-tuned model Mixtral 8×7B in this study setup showed even less rater-identified inconsistencies than the larger LLM Mixtral 8×22B with ICL, which highlights the capacity of domain alignment to reduce hallucination and narrows the model’s behavior to task relevant outputs.

In contrast, models based on zero-shot or few-shot prompting showed greater variability in output quality. For example, the Mixtral 8×7B with zero-shot prompting underperformed in metrics related to flow and organization (succinctness, syntheticism, and structural organization), echoing concerns from earlier studies regarding prompt-based methods lacking domain-specific refinement [25,26].

Interestingly, despite the larger model size, the LLaMA 3.1 70B model did not consistently outperform the smaller fine-tuned Mixtral model—corroborating recent findings that model size alone does not guarantee clinical output quality [27,28].

Further, case complexity had a clear impact on ratings. DN for simple cases were rated an average of 3 points higher than those for complex cases, regardless of the model used. This indicates that LLMs remain in general more capable of generating accurate summaries for straightforward scenarios, while struggling to synthesize multifaceted histories or nuanced reasoning chains—limitations echoed in earlier clinical NLP evaluations [29,30].

Finally, the Turing Test evaluation provided additional interesting insights into the realism of the generated DNs. Individual rater accuracies were compared against chance-level performance using 2-sided exact binomial tests. To account for multiple testing across raters, Bonferroni-adjusted *P* values were calculated. After correction, none of the individual raters showed classification accuracy significantly different from chance-level performance. The observed nondistinguishability for the discrimination of generated versus human-written DN underscores the maturity of generative AI models in replicating stylistic and structural features of clinical documentation. Notably, the 2 fellows and 2 senior clinicians’ classification accuracy was near chance level, highlighting the increasing indistinguishability of synthetic clinical text in the fine-tuned setup. This result underscores the potential of language models to produce DNs with a layout and stylistic quality very similar to those authored by humans. Without deep contextual knowledge of patient histories, even experienced clinicians fail to assess the realism of generated DN.

Together, these findings suggest that domain-specific fine-tuning, structured input formats, and clinical supervision are critical to achieve high-quality AI-generated documentation and further safeguards are necessary, particularly when applying models to complex medical contexts in clinical setups raising additional important regulatory considerations.

From an ethical perspective, the high stylistic similarity between AI-generated and clinician-authored DN raises important concerns regarding transparency and accountability. Clinicians, downstream care providers, and patients themselves may not be able to reliably distinguish between AI-generated and human-authored content, potentially leading to overreliance in automatically generated documentation. Therefore, AI-generated DNs should be subject to mandatory clinician review, with ultimate responsibility remaining with the treating physician in accordance with the clinician-in-the-loop paradigm. Clear governance frameworks, transparency regarding AI use, and robust human oversight are essential to ensure patient safety and support the responsible implementation of AI in clinical documentation.

### Limitations

Some limitations should be acknowledged. First, although each discharge summary was evaluated by 9 physicians, inter-rater agreement was low, indicating substantial variability in the perception of clinical quality even among trained professionals.

Second, while SFT improved model performance, hallucination errors and factual completeness were assessed through direct comparison with available clinical information but were not systematically quantified beyond subjective scoring using the mPDQI-9 framework. Future work should incorporate structured or qualitative annotation to more rigorously evaluate clinical accuracy.

Furthermore, the study was performed under computational resource constraints that reflect the infrastructure commonly available in current hospital environments. While this limited the use of larger or more computationally intensive models, it reflects the real-world conditions under which clinical AI systems must operate and thereby enhances the practical relevance of our findings.

Finally, real-world clinical outcomes such as time saved in documentation or improvements in patient comprehension were not assessed and will be investigated in ongoing deployment studies.

## Conclusion

This study demonstrates that fine-tuned LLMs deployed within secure, privacy-compliant hospital infrastructures can generate clinically acceptable DN for inpatients. Among all tested configurations, the Mixtral 8×7B model with SFT achieved the best results in both calculated metrics and physician evaluations, particularly in narrative style and structure. However, performance declined with increasing case complexity.

While generative AI may offer great potential as a support tool for documentation, its impact on clinician workload, decision-making, and patient outcomes was not yet assessed within this study. In addition, the high stylistic similarity between AI-generated and human-authored text raises ethical and safety considerations, particularly if outputs are not clearly distinguishable, necessitating robust safeguards for transparency, accountability, and clinical oversight.

Overall, the results demonstrate the feasibility of generating structured discharge summaries using locally deployed LLMs in a privacy-compliant setting. Future work should focus on improving robustness in complex cases, developing structured approaches for hallucination and safety evaluation, and prospectively validating performance and utility in real-world clinical workflows.

## Data Availability

The datasets analyzed in this study are not publicly available due to institutional patient privacy regulations and the inclusion of complete EHRs. However, we welcome collaboration with other institutions through the corresponding author. Data exchange may be possible under appropriate data transfer agreements and with relevant ethical approvals. The notebooks and prompts used in this study are publicly available on GitLab (https://gitlab.com/public-ksa/dngenai/dngenai).
