# Supplementary material for: Toward Responsible and Privacy-Preserving Generation of Inpatient Clinical Discharge Notes Using Large Language Models
Source: Comput Struct Biotechnol J. 2026 Jul 9;35(2):0151. doi: 10.34133/csbj.0151 (PMC13632913; doi:10.34133/csbj.0151)
Supplement: Supplementary file 1 [file csbj.0151.f1.pdf]

Supplements

English Translation of Original German Prompt

As the attending physician, you are tasked with writing the “Assessment and Course” section of a discharge report in German. Use only the information provided in JSON format (medical history, list of diagnoses) and create a concise, medically sound narrative summary. Focus strictly on explicitly stated medical facts and avoid any additional or speculative content. The text should be objective, precise, and well-structured, without unnecessary details or repetition.

Emphasize interventions or therapeutic medication administration, rather than radiological findings. Avoid bullet points or enumerations. Use appropriate medical terminology as commonly used in clinical practice. Summarize only those diagnoses that were made during the current inpatient stay (from admission to discharge). Information outside this time frame is irrelevant and should not be mentioned. Each diagnosis has an assigned number that you can refer to in the report. Ensure the text is complete and captures the relevant course of events, avoiding abrupt endings or premature cutoffs especially not immediately after a date.

Figure S1: Conception of prompting used to generate discharge summaries in Zero-shot and ICL settings in English (translated from original prompting).

Original German Prompt

Als leitender Arzt bist du beauftragt, den Abschnitt ’Beurteilung und Verlauf’ eines Austrittsberichts auf Deutsch zu verfassen. Nutze ausschliesslich die als JSON bereitgestellten Informationen (Anamnese, Diagnoseliste) und erstelle eine prägnante, medizinisch fundierte Zusammenfassung im Fliesstext. Beschränke dich auf explizit genannte medizinische Fakten und vermeide zusätzliche oder spekulative Angaben.

Der Text soll sachlich, präzise und strukturiert sein, ohne unnötige Details oder Wiederholungen. Fokussiere dich auch auf Interventionen oder therapeutische Medikamentengaben und weniger auf radiologische Befunde. Verzichte auf Listen oder Aufzählungen. Verwende angemessenes medizinisches Fachvokabular, wie es in der klinischen Praxis üblich ist.

Fasse nur diejenigen Diagnosen zusammen, die während des aktuellen stationären Aufenthalts (vom Eintrittsdatum bis zum Austrittsdatum) gestellt wurden. Dinge ausserhalb dieses Zeitraums sind nicht relevant, erwähne sie daher nicht. Die Diagnosen haben eine zugewiesene Nummer, auf die du dich im Bericht beziehen kannst. Stelle sicher, dass der Text vollständig ist und den relevanten Verlauf zusammenfasst, ohne abrupt zu enden oder vorzeitig abzubrechen, insbesondere nicht nach einem Datum.

Figure S2: Conception of prompting used to generate discharge summaries in Zero-shot and ICL settings in German.

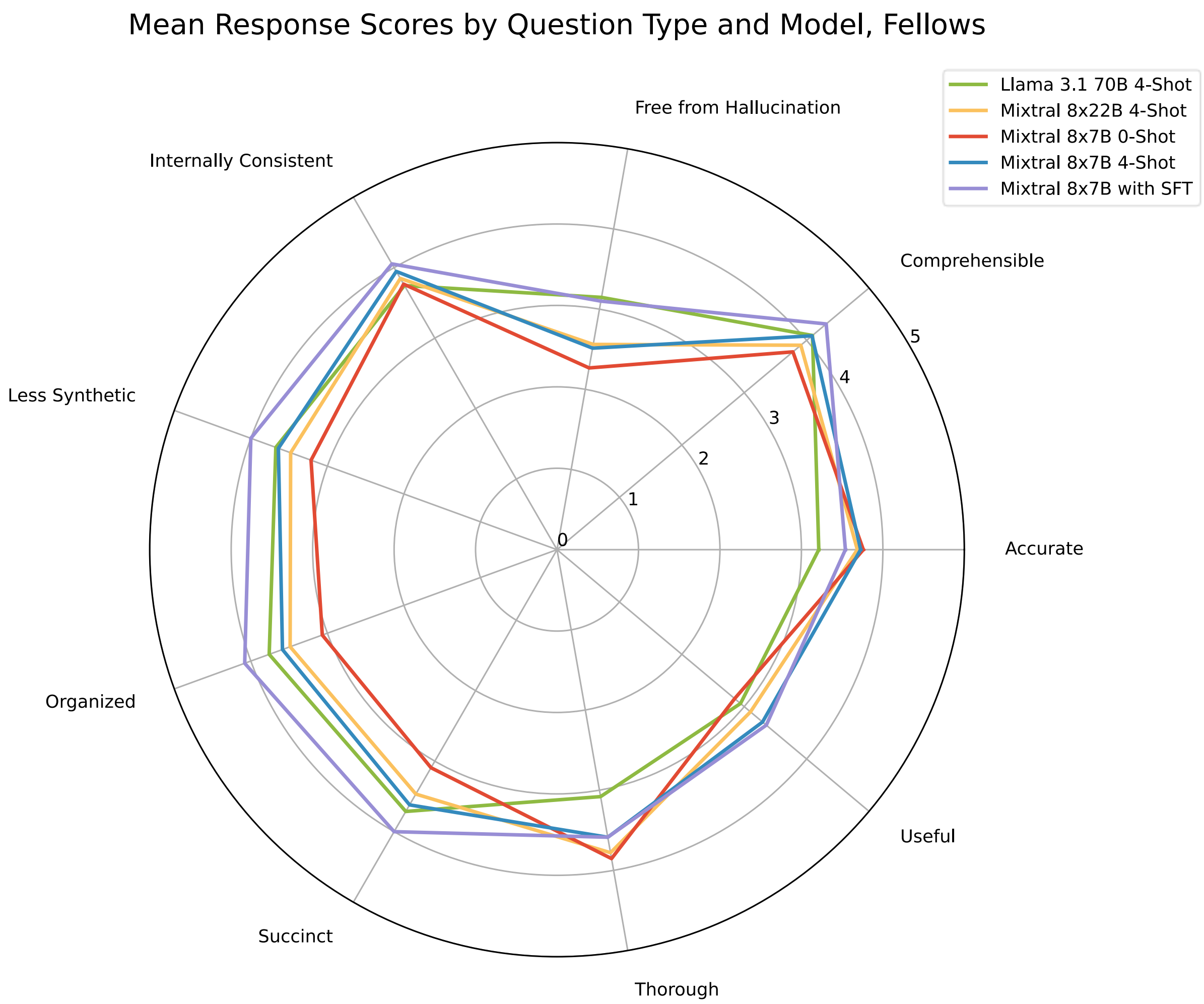

Figure S3: Mean Fellows Response Scores by Question Type and Model

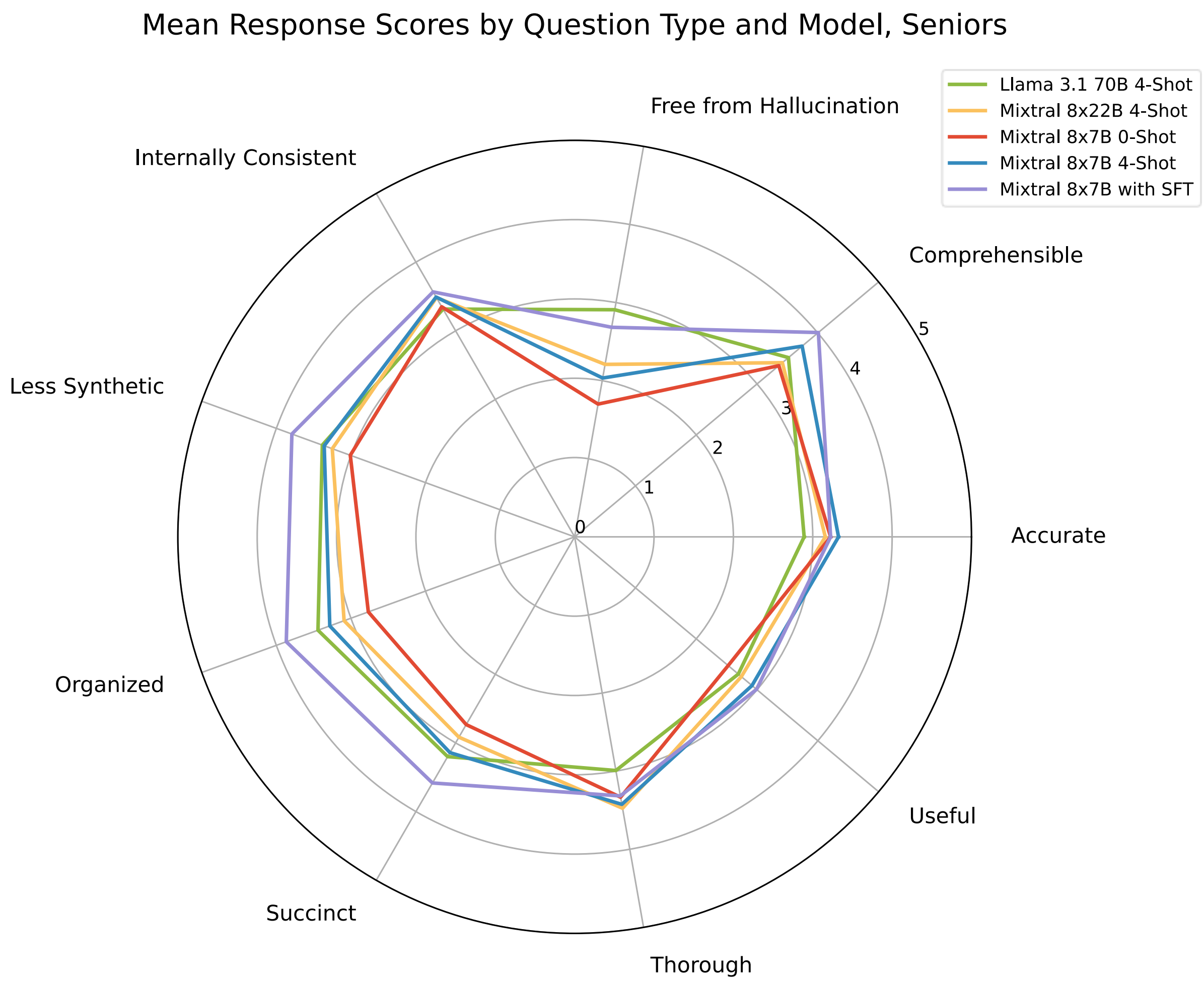

Figure S4: Mean Senior Response Scores by Question Type and Model

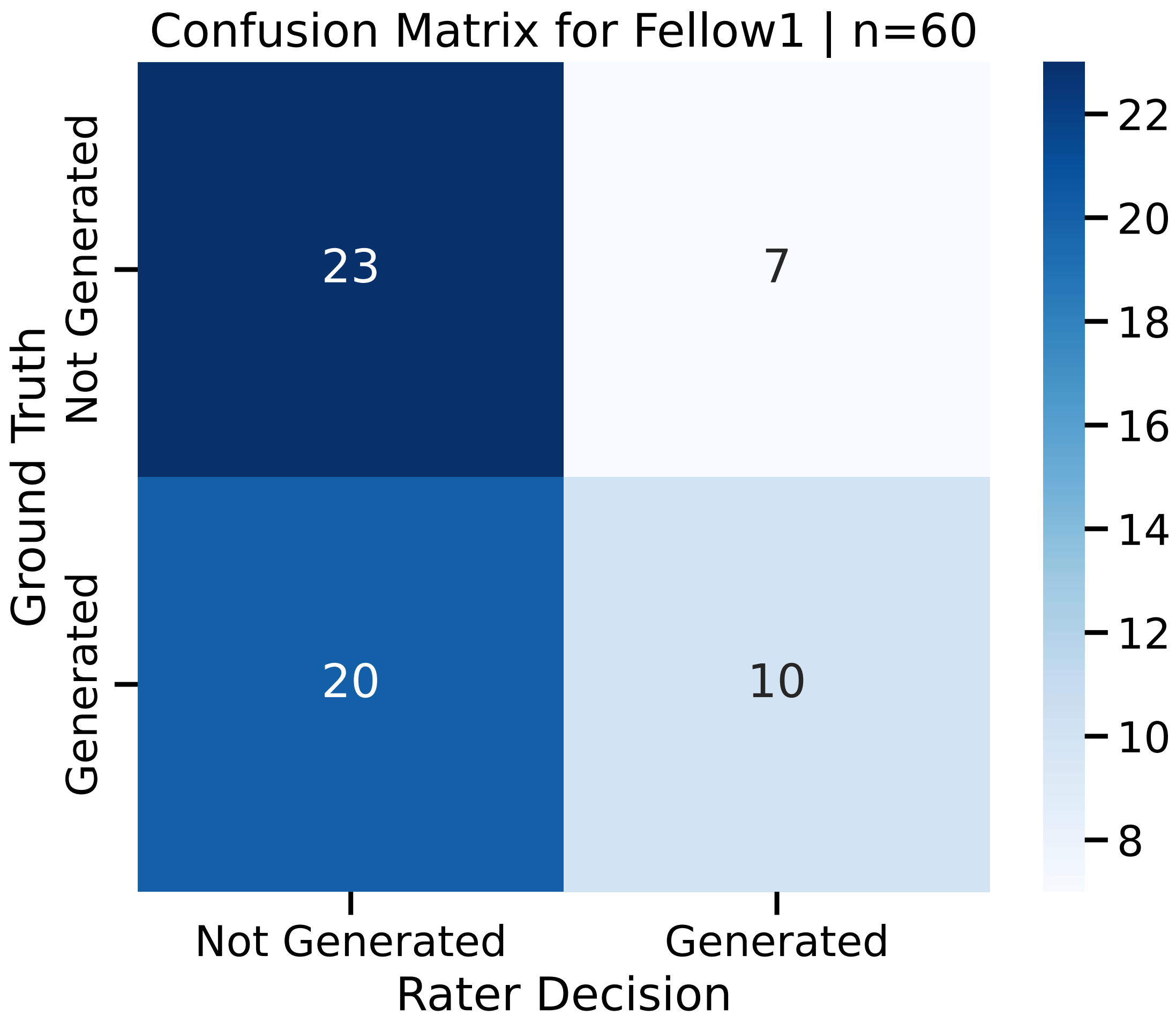

Figure S5: Turing test: Fellow physician No. 1 distinguishing AI-generated from human authored DN.

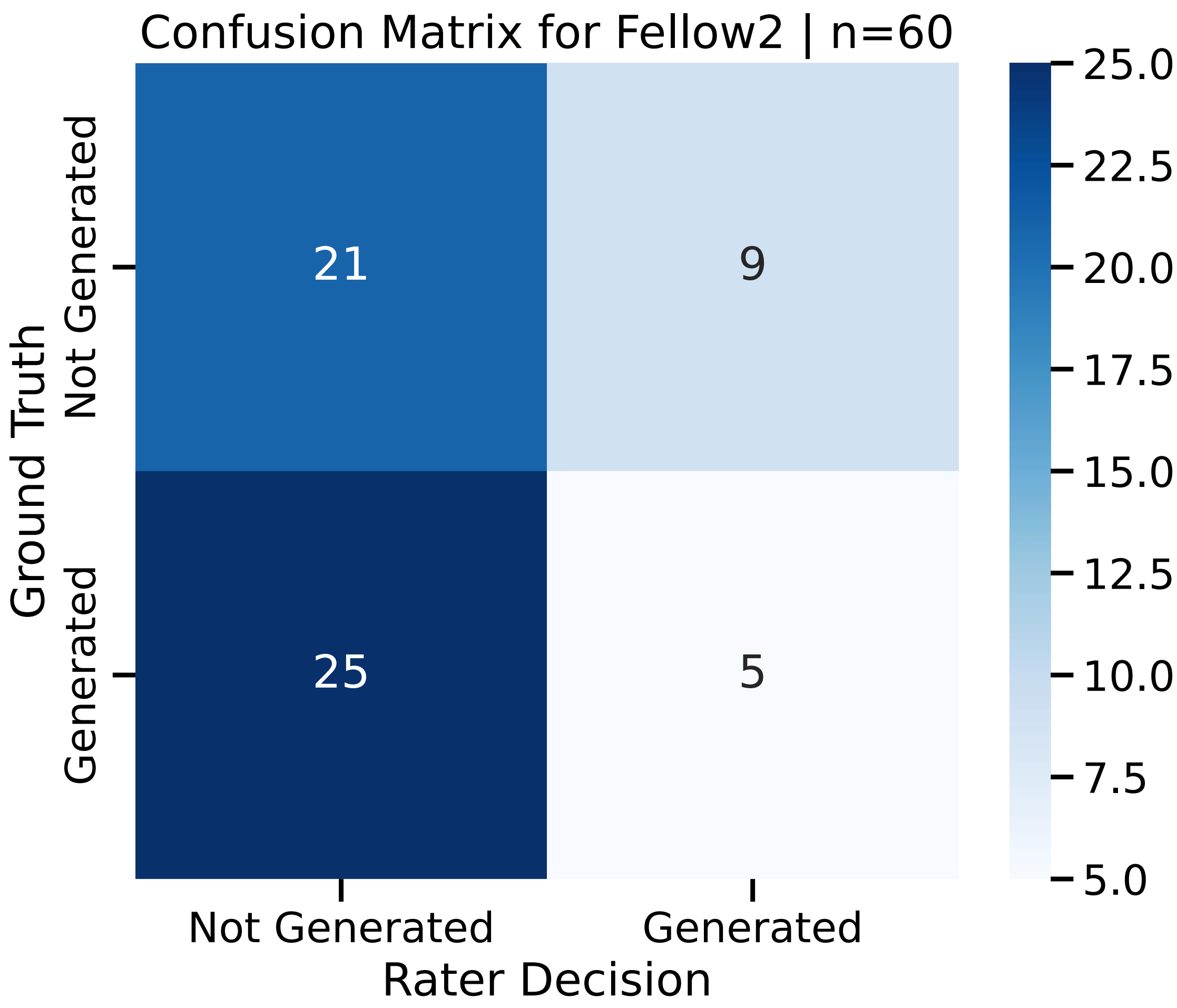

Figure S6: Turing test: Fellow physician No. 2 distinguishing AI-generated from human authored DN.

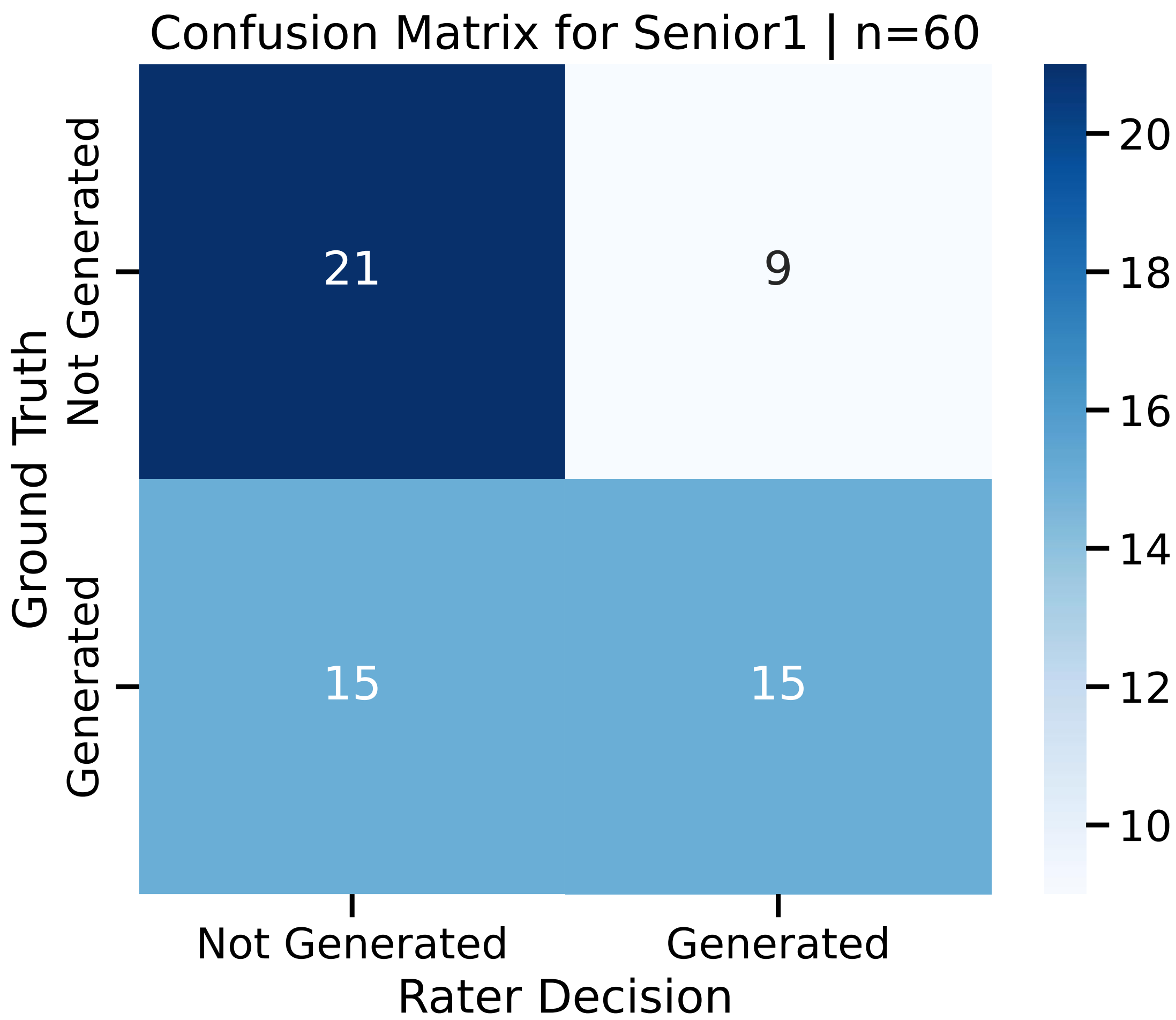

Figure S7: Turing test: Senior physician No. 1 distinguishing AI-generated from human authored DN.

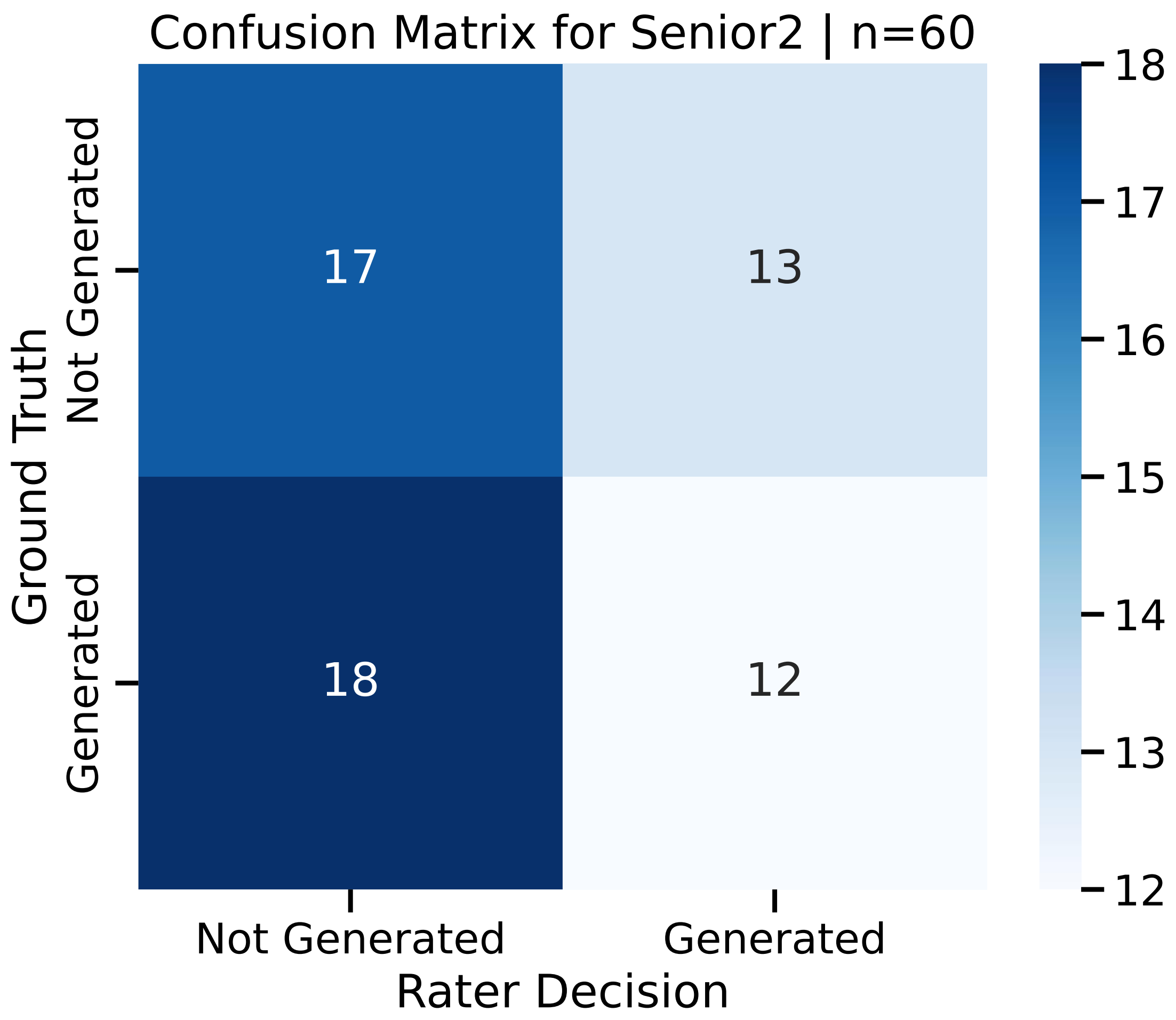

Figure S8: Turing test: Senior physician No. 2 distinguishing AI-generated from human authored DN.
